# Reduced CV risk with long-term GH replacement in AGHD: data from two large observational studies

**DOI:** 10.1530/EC-22-0267

**Published:** 2022-12-14

**Authors:** Charlotte Höybye, Beverly M K Biller, Jean-Marc Ferran, Murray B Gordon, Nicky Kelepouris, Navid Nedjatian, Anne H Olsen, Matthias M Weber

**Affiliations:** 1 Department of Endocrinology and Department of Molecular Medicine and Surgery, Karolinska University Hospital and Karolinska Institute, Stockholm, Sweden; 2 Neuroendocrine Unit, Massachusetts General Hospital, Massachusetts General Hospital, Boston, Massachusetts, USA; 3 Qualiance ApS, Copenhagen, Denmark; 4 Allegheny Neuroendocrinology Center, Division of Endocrinology, Allegheny General Hospital, Pittsburgh, Pennsylvania, USA; 5 US Medical Affairs-Rare Endocrine Disorders, Novo Nordisk, Inc, Plainsboro, New Jersey, USA; 6 Global Medical Affairs – Rare Endocrine Disorders, Novo Nordisk Health Care AG, Zurich, Switzerland; 7 Epidemiology, Novo Nordisk A/S, Soborg, Denmark; 8 Unit of Endocrinology, 1, Medical Department, University Hospital, Universitätsmedizin Mainz, der Johannes Gutenberg-Universität, Mainz, Germany

**Keywords:** norditropin, growth hormone, cardiovascular risk, NordiNet, ANSWER

## Abstract

Adult growth hormone deficiency (AGHD) is associated with an increased risk of cardiovascular (CV) disease. Long-term growth hormone (GH) treatment could improve CV outcomes. The objective of this study was to evaluate CV disease risk in patients with AGHD who received GH replacement therapy for up to 10 years as part of NordiNet^®^ IOS (NCT00960128) and the ANSWER Program (NCT01009905). The studies were observational, non-interventional and multicentre, monitoring long-term effectiveness and safety of GH treatment. NordiNet^®^ IOS involved 23 countries (469 sites) across Europe and the Middle East. The ANSWER Program was conducted in the USA (207 sites). This analysis included patients aged 18–75 years who were GH naïve at study entry, who had ≤10 years of GH treatment data and who could be assessed for CV risk for at least 1 follow-up year. The main outcome measure was risk of CV disease by age 75 years, as calculated with the Multinational Cardiovascular Risk Consortium model (Brunner score) using non-high-density lipoprotein cholesterol adjusted for age, sex and CV risk factors. The results of this analysis showed that CV risk decreased gradually over the 10-year period for GH-treated patients. The risk was lower for patients treated for 2 and 7 years vs age- and sex-matched control groups (not yet started treatment) (14.51% vs 16.15%; *P* = 0.0105 and 13.53% vs 16.81%; *P* = 0.0001, respectively). This suggests that GH treatment in people with AGHD may reduce the risk of CV disease by age 75 years compared with matched controls.

## Introduction

Adult growth hormone deficiency (AGHD) is associated with increased visceral adiposity, insulin resistance, dyslipidaemia and hyperglycaemia (1). AGHD may persist from childhood or be acquired in adulthood as a result of pituitary adenomas and their related therapies, brain trauma or other hypothalamic-pituitary disorders (2, 3). Furthermore, patients with AGHD typically have unfavourable alterations in body composition, a decreased capacity for exercise, impaired quality of life, an adverse lipid profile and an increased risk for cardiovascular (CV)-associated disease, diabetes and metabolic syndrome vs healthy adults (4, 5, 6, 7).

The use of growth hormone (GH) replacement therapy in AGHD has been shown to improve body composition (reduced fat mass, increased lean mass and increased muscle strength), bone mineral density and CV risk markers (increased high-density lipoprotein (HDL) cholesterol and reductions in low-density lipoprotein (LDL) cholesterol, C-reactive protein, diastolic blood pressure and carotid intima-media thickness), as well as reducing CV risk estimates and improving patients’ quality of life (5, 8, 9). Although GH replacement therapy may initially impair glucose metabolism, long-term GH treatment can lead to improved insulin levels and glycaemia due to concomitant body composition changes (10, 11, 12). Additionally, replacement with other hormones, especially pharmacological doses of glucocorticoids, might have a negative impact on insulin sensitivity (13).

Previously described models estimate the long-term probabilities for CV disease events based on the presence of CV risk factors, including cholesterol concentration, body mass index (BMI), systolic blood pressure, smoking status and familial history (14, 15). The Multinational Cardiovascular Risk Consortium model described by Brunner *et al.* estimates the probabilities of a CV disease event by the age of 75 years using non-HDL cholesterol categories defined according to thresholds described in European guidelines, adjusted for age, sex and presence of classical modifiable CV risk factors (14). The model was developed using individual-level data and excluded participants with prevalent CV disease (14).

The objective of this study is to evaluate the risk of having CV disease by the age of 75 years, calculated using the Multinational CV Risk Consortium model (Brunner score) (14), in patients receiving long-term GH replacement therapy for AGHD (GH treated) who were enrolled in two non-interventional, multicentre studies: the NordiNet^®^ International Outcome Study (IOS) and the American Norditropin^®^ Studies: Web-Enabled Research (ANSWER) Program (16, 17). The CV risks of patients after treatment initiation were compared with the CV risks of age- and sex-matched control groups (matched control) consisting of patients with AGHD at study enrolment of the NordiNet^®^ IOS or the ANSWER Program who had not yet begun GH replacement therapy and can be considered as untreated.

## Materials and methods

### Study design

The detailed NordiNet^®^ IOS (NCT00960128) and ANSWER Program (NCT01009905) study designs and methods have previously been reported (16, 17). The NordiNet^®^ IOS took place from April 2006 to December 2016, involved 23 countries (469 sites) across Europe and the Middle East and included 2524 adults (17). The ANSWER Program was conducted between June 2002 and September 2016 in the USA (207 sites) and included 982 adults (17). Both studies used the same electronic data-management platform (NordiNet^®^/NovoNet^®^) and were complementary with similar aims. They were approved by the relevant ethics committees, conducted with written consent from patients and pseudonymisation of all data and performed in accordance with the Declaration of Helsinki, regulatory requirements and Guideline for Good Pharmacoepidemiology Practices.

### Patient population

In this analysis, we used pooled data from GH-treatment-naïve (GH-naïve) patients aged 18–75 years from the full analysis set (FAS) of the NordiNet^®^ IOS and ANSWER Program, with up to 10 years of GH treatment data, for whom the CV risk could be assessed for at least one of the 10 follow-up years. Only GH-naïve patients from the FAS were included in this analysis; further details of the FAS are described in the Supplementary Appendix.

**Figure 1:**
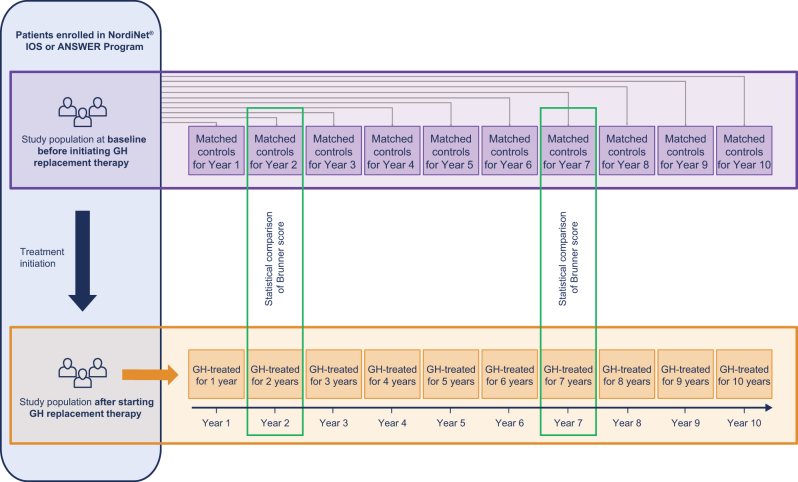
Study design. At each follow-up year, GH-treated patients were compared to age- and sex-matched untreated patients from the control group (taken from baseline). GH, growth hormone; IOS, International Outcome Study.

The risk of having CV disease at the age of 75 years was calculated for each year of follow-up for GH-treated patients with sufficient data for calculating the CV risk on a given year (Fig. 1) (see the statistical analysis section for further details). Due to the nature of observational studies, there were missing data which resulted in different numbers of treated patients in each follow-up year.

For each follow-up year of GH replacement therapy, we used the CV risk of an age- and sex-matched control group of patients with AGHD at study enrolment (of either study) before GH replacement initiation (Fig. 1) as a comparator. A 2:1 ratio (control group:patients) for matching was used. The control groups were different for each of the follow-up years, as they were cross-sectional cohorts chosen to match the GH-treated patients in each of those follow-up years. No longitudinal follow-up was feasible for untreated patients from the matched control groups, as they all started on GH replacement after entering the NordiNet^®^ IOS or ANSWER Program.

### Variables and statistical analysis

The estimated risk of having CV disease by age 75 years was calculated using the Multinational CV Risk Consortium model, as described by Brunner *et al.* (14). The Brunner score categorises patients by sex, age (<45, 45 to <60 and 60–75 years), non-HDL cholesterol levels (<2.6 mmol/L, 2.6–<3.7 mmol/L, 3.7 to <4.8 mmol/L, 4.8 to <5.7 mmol/L and ≥5.7 mmol/L) and the number of other CV risk factors (hypertension, daily smoking, diabetes and obesity; 0–1, ≥2) (Supplementary Fig. 1, see section on supplementary materials given at the end of this article). Standard definitions of non-HDL cholesterol, hypertension, smoking and diabetes were used (details are in the Supplementary Appendix). Measurements in both the NordiNet^®^ IOS and ANSWER Program studies were not conducted in a central laboratory; parameters were measured according to standard clinical practice.

For patients classified as having 0–1 risk factor(s), data had to be available for a given year for at least three risk factors and not be positive for any, or data had to be available for all four risk factors and be positive for only one. Patients classified as having ≥2 risk factors must have had data available for a given year for at least two risk factors and be positive for at least two. Comparison of GH-treated vs matched control patients was cross-sectional only, as the data used for the control groups were taken during study enrolment (NordiNet^®^ IOS or ANSWER Program) before patients started GH treatment. Longitudinal comparison between the GH-treated patients and the matched control groups could not be performed, as all patients eventually received GH treatment. The analyses of change in Brunner score from baseline to specific follow-up years for GH-treated patients and various subgroups (sex and number of pituitary deficiencies) were longitudinal.

The estimated risk of having CV disease by age of 75 years (Brunner score) after 2 (shorter term) and 7 years (longer term) of GH replacement therapy for the GH-treated patients was calculated and compared with the CV risk of the respective matched control group. A Welch–Satterthwaite *t*-test was performed due to unequal variances. Planned statistical comparison was conducted for two time points (Year 2 and Year 7) to avoid multiple comparisons bias. Regression analysis was performed to investigate a trend in change from baseline (study enrolment) for Brunner score and years of treatment (Years 1–10) and confirmed via a non-parametric test (Jonckheere–Terpstra). At Year 2 and Year 7, we also descriptively analysed the parameters used to calculate Brunner score (non-HDL cholesterol category, obesity, hypertension, diabetes and identification as a daily smoker).

Two sets of sensitivity analyses were conducted for Years 2 and 7 of Brunner score comparisons between GH-treated and matched control patients. One analysis only included patients with data available for three out of four risk factor assessments, and the second analysis only included patients with data available for four out of four risk factor assessments.

### Matching algorithm for control groups

For each follow-up year, GH-treated patients with a Brunner score were randomly matched with up to two control patients from the FAS (at study enrolment) (Fig. 1) who had a Brunner score calculated using their baseline data points before initiating the GH replacement therapy. They were matched based on their sex and age (±0.5 years for Year 1, ±1 year otherwise). For some patients, <2 matched control patients could be identified for a given year, as, in some incidences, no suitable match was identified for a selected treated patient in a given year.

As the Brunner score reflects the risk of CV disease at a fixed point in time in the future (age of 75 years), the score is, by definition, expected to decrease gradually with age because, as patient age increases, the duration of exposure to risk factors before reaching the age of 75 years is shortened. For example, assuming the same level of risk factors, a 40-year-old person has greater risk of developing CV disease by the age of 75 years vs a 73-year-old person. Therefore, it was important to adjust for the effect of age and to compare treated patients with an age- and sex-matched (untreated) control group.

## Results

### Patient population

This analysis included 708 adults with AGHD (18–75 years of age) who were GH naïve at baseline with up to 10 years of GH treatment follow-up data for whom Brunner score could be assessed for at least one of the follow-up years. Their baseline characteristics are presented in Table 1. Of the 708 patients, 534 (75.4%) had 2–5 pituitary deficiencies (multiple pituitary hormone deficiency (MPHD)) reported, including GHD, and 174 patients (24.6%) had only 1 pituitary deficiency reported (presumed isolated GHD (IGHD)) (Table 1). We considered the latter group as having presumed IGHD, as the presence of other pituitary deficiencies at baseline could not be excluded. Most patients (*n*  = 661, 93.4%) acquired GHD during adulthood, with only 47 (6.6%) with a diagnosis from childhood (Table 1). Of the 608 patients with a BMI recording at baseline (mean 28.8, s.d. 6.2), 35.2% had BMI above 30 kg/m^2^. We did not observe any difference in baseline BMI between patients with presumed IGHD and MPHD (mean (s.d.), 28.2 (6.3) and 29.0 (6.1), respectively). Additionally, no difference was observed in the percentage of patients with obesity between the presumed IGHD and MPHD groups (31.7% and 36.3%, respectively; *P* = 0.3665). The aetiology of GHD was predominantly due to pituitary adenoma (67.7%) or its treatment (Table 1). A small number of patients had acromegaly (*n*  = 9) or Cushing’s syndrome (*n*  = 12). In total, 22 (3.1%) patients were reported as taking concomitant lipid-lowering medications, 273 patients (38.6%) were reported to be taking thyroid hormone and 322 patients (45.4%) were reported as taking corticosteroids at baseline and/or during the follow-up years.

**Table 1:** Baseline demographics and clinical characteristics of all patients included in this analysis (GH-naïve patients, at study enrolment, aged 18–75 years with up to 10 years of GH treatment data for whom the CV risk could be assessed for at least one of the follow-up years).

Characteristic	*N* = 708
Female, *n* (%)	317 (44.7)
Mean age, years (s.d.) (*n* = 683)	47.0 (14.0)
Mean IGF-I SDS (s.d.) (*n* = 554)	−1.16 (1.43)
Mean BMI, kg/m^2^ (s.d.) (*n* = 608)	28.8 (6.2)
Number of pituitary deficiencies, *n* (%)	
1	174 (24.6)
2	132 (18.6)
3	98 (13.8)
4	203 (28.7)
5	101 (14.3)
Onset of GHD, n (%)	
Childhood	47 (6.6)
Adult	661 (93.4)
Aetiology of GHD, *n* (%)	
Pituitary adenoma or its treatment	479 (67.7)
Unknown	76 (10.7)
Post-procedural hypopituitarism	70 (9.9)
Craniopharyngioma or its treatment	27 (3.8)
Traumatic brain injury	15 (2.1)
Congenital	14 (2.0)
Sheehan syndrome	12 (1.7)
Other acquired causes	10 (1.4)
Isolated/idiopathic	5 (0.7)
Patients with risk factors, *n* (%)	
Obesity	214 (30.2)
Missing	100 (14.1)
Hypertension	166 (23.5)
Missing	141 (19.9)
Diabetes	51 (7.2)
Missing	202 (28.5)
Identified as a daily smoker	4 (0.6)
Missing	677 (95.6)
Non-HDL cholesterol category, *n* (%)	
Missing	239 (33.8)
<2.6 mmol/L	35 (4.9)
2.6 to >3.7 mmol/L	118 (16.7)
3.7 to >4.8 mmol/L	165 (23.3)
4.8 to >5.7 mmol/L	91 (12.9)
≥5.7 mmol/L	60 (8.5)

Patients were identified as hypertensive when their SBP was ≥140 mmHg or when their SBP was <140 mmHg with reported concomitant use of an antihypertensive medication. Patients were considered positive for daily smoking if they were identified as a regular smoker in the lifestyle section of the study case reports. Patients were identified as having diabetes when their FPG was ≥126 mg/dL, or their HbA1c was ≥47.5 mmol/mol (6.5%) or when either FPG or HbA1c was below the defined threshold with reported concomitant use of antidiabetic medications. Patients were identified as obese if their BMI was ≥30 kg/m^2^.

BMI, body mass index; CV, cardiovascular; FPG, fasting plasma glucose; GH, growth hormone; GHD, growth hormone deficiency; HbA1c, glycated haemoglobin; HDL, high-density lipoprotein; IGF-I, insulin-like growth factor-I; *n/N*, number of participants; SBP, systolic blood pressure.

Mean (s.d.) duration of exposure was 5.2 (4.3) years for females and 5.7 (4.7) years for males in the NordiNet^®^ IOS population and 3.7 (3.4) years for females and 3.7 (3.6) for males in the ANSWER Program population (both FAS). A total of 265 GH-treated patients with a CV risk score at Year 2 were compared with 285 matched control patients, and 110 GH-treated patients with CV risk score at Year 7 were compared with 178 matched control patients. Their characteristics are presented in Table 2.

**Table 2:** Patient characteristics of patients included in statistical comparison of GH-treated and matched control patients at Year 2 and Year 7.

	Year 2		Year 7	
	GH treated	Matched control group^a^	GH treated	Matched control group^a^
Number of patients, *n*	265	285	110	178
Female, *n* (%)	118 (44.5)	126 (44.2)	46 (41.8)	72 (40.5)
Mean age, years (s.d.)	49.5 (13.7)	47.6 (13.8)	52.9 (12.9)	51.7 (12.3)
Mean GH dose, mg/day (s.d.)	0.32 (0.22) (*n* = 258)	–	0.36 (0.17) (*n* = 104)	–
Mean IGF-I SDS	0.45 (1.33) (*n* = 260)	−1.25 (1.46) (*n* = 274)	0.42 (1.14) (*n* = 109)	−1.05 (1.40) (*n* = 172)
Mean BMI, kg/m^2^ (s.d.)	29.2 (6.4) (*n* = 259)	28.5 (5.9) (*n* = 285)	29.2 (6.8) (*n* = 107)	29.0 (5.6) (*n* = 178)
Onset of GHD, *n* (%)				
Childhood	12 (4.5)	16 (5.6)	7 (6.4)	4 (2.3)
Adult	253 (95.5)	269 (94.4)	103 (93.6)	174 (97.8)
Patients with risk factors, *n* (%)				
Obesity	96 (36.2)	88 (30.9)	33 (30.0)	61 (34.3)
Missing	6 (2.3)	0 (0.0)	3 (2.7)	0 (0.0)
Hypertension	74 (27.9)	77 (27.0)	34 (30.9)	55 (30.9)
Missing	8 (3.0)	4 (1.4)	4 (3.6)	1 (0.6)
Diabetes	33 (12.5)	37 (13.0)	15 (13.6)	24 (13.5)
Missing	6 (2.3)	10 (3.5)	3 (2.7)	9 (5.1)
Identified as a daily smoker	16 (6.0)	3 (1.1)	7 (6.4)	2 (1.1)
Missing	71 (26.8)	260 (91.2)	19 (17.3)	162 (91.0)
Non-HDL cholesterol category, *n* (%)				
<2.6 mmol/L	16 (6.0)	18 (6.3)	7 (6.4)	10 (5.6)
2.6 to >3.7 mmol/L	92 (34.7)	74 (26.0)	49 (44.6)	39 (21.9)
3.7 to >4.8 mmol/L	99 (37.4)	96 (33.7)	35 (31.8)	60 (33.7)
4.8 to >5.7 mmol/L	36 (13.6)	61 (21.4)	15 (13.6)	39 (21.9)
≥5.7 mmol/L	22 (8.3)	36 (12.6)	4 (3.6)	30 (16.9)

Patients were identified as hypertensive when their SBP was ≥140 mmHg or when their SBP was <140 mmHg with reported concomitant use of an antihypertensive medication. Patients were considered positive for daily smoking if they were identified as a regular smoker in the lifestyle section of the study case reports. Patients were identified as having diabetes when their FPG was ≥126 mg/dL, or their HbA1c was ≥47.5 mmol/mol (6.5%) or when either FPG or HbA1c was below the defined threshold with reported concomitant use of antidiabetic medications. Patients were identified as obese if their BMI was ≥30 kg/m^2^.

^a^Age- and sex-matched untreated patients at study enrolment of NordiNet^®^ IOS or the ANSWER Program before initiating GH treatment.

BMI, body mass index; FPG, fasting plasma glucose; GH, growth hormone; GHD, growth hormone deficiency; HbA1c, glycated haemoglobin; HDL, high-density lipoprotein; IGF-I, insulin-like growth factor-I; IOS, International Outcome Study; *n*, number of participants; SBP, systolic blood pressure.

### IGF-I levels in GH-treated patients and matched control groups

**Figure 2:**
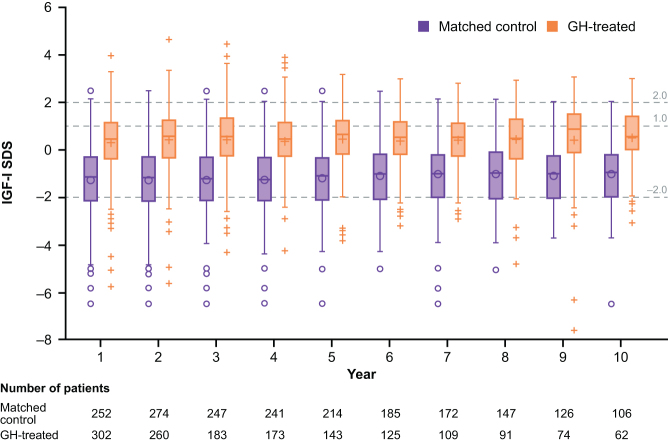
Cross-sectional comparison of IGF-I SDS between GH-treated patients at a follow-up year vs matched (age and sex) control groups. Shown are the mean (noughts/crosses within the box), median (dash within the box), 25th/75th percentiles (box) and maximum overserved values (noughts and crosses above the box). GH, growth hormone; IGF-I, insulin-like growth factor-I; SDS, standard deviation score.

The mean (s.d.) baseline insulin-like growth factor-I (IGF-I) SDS of GH-treated patients included in this analysis was −1.16 (1.43). In patients who were treated for 1 year, IGF-I SDS was considerably higher than baseline with a mean (s.d.) of 0.33 (1.33). In GH-treated patients, mean IGF-I SDS levels were similar for the 10 years of follow-up, ranging from 0.33 to 0.54, with the higher level observed in Year 10 (Fig. 2). In the matched control groups, the mean IGF-I SDS was consistently approximately −1.0 SDS (range: −0.96 to −1.25) (Fig. 2). Female patients (in both GH-treated and matched control groups) had a slightly lower IGF-I SDS vs male patients at baseline and during the follow-up years (Supplementary Fig. 2). It was not possible to determine the correlation between IGF-I SDS and Brunner score due to an insufficient number of patients with both a Brunner score and an IGF-I SDS measurement at Years 2 and 7.

### Comparing the risk of CV disease between GH-treated patients and matched control groups using the Brunner score

**Figure 3:**
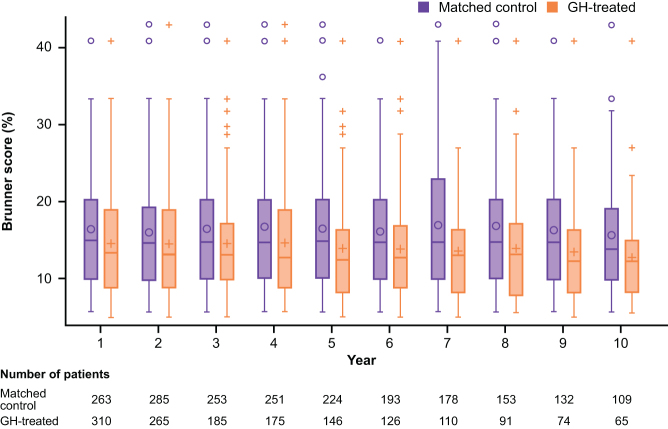
Cross-sectional comparison of Brunner score between GH-treated patients at a follow-up year vs matched (age and sex) control groups. Shown are the mean (noughts/crosses within the box), median (dash within the box), 25th/75th percentiles (box) and maximum overserved values (noughts and crosses above the box). GH, growth hormone.

The estimated risk of having CV disease by age 75 years in GH-treated patients and matched control groups is shown in Table 3 and Fig. 3. A lower Brunner score indicates lower risk. The Brunner scores for GH-treated patients were lower than the values for the matched control groups throughout the 10 years of follow-up (Table 3). As the number of follow-up years increased, the Brunner score for GH-treated patients reduced from 16.04% at baseline to 12.69% at Year 10. However, for matched control group patients, the scores were similar throughout, ranging from 16.19 to 16.81% (Table 3).

**Table 3:** Brunner risk score.

GH-treated patients				Matched control group^a^			GH-treated patients with baseline and follow-up data		
Years of treatment	*n*	Brunner risk score, mean %	Brunner risk score, s.d. %	*n*	Brunner risk score, mean %	Brunner risk score, s.d. %	*n*	Relative change from baseline, mean %	Relative change from baseline, s.d. %
0	301	16.04	8.02	–	–	–	–	–	–
1	310	14.66	6.97	263	16.30	7.96	123	−1.26	21.34
**2**^**b**^	**265**	**14.51**	**6.90**	**285**	**16.15**	**8.07**	**115**	−3.54	**23.24**
3	185	14.59	6.90	253	16.71	8.18	75	−4.11	25.27
4	175	14.66	7.52	251	16.60	8.35	64	−5.58	27.18
5	146	13.95	7.30	224	16.39	7.30	57	−6.09	25.30
6	126	13.78	6.61	193	16.19	7.57	46	−12.40	26.07
**7**^**b**^	**110**	**13.53**	**6.13**	**178**	**16.81**	**8.18**	**45**	−14.40	**27.65**
8	91	13.97	7.24	153	16.61	8.37	29	−19.10	21.31
9	74	13.40	6.48	132	16.51	8.72	20	−15.00	19.14
10	65	12.69	6.30	109	16.27	7.69	15	−4.77	31.74

The risk of having CV disease by age 75 years was calculated using the Multinational CV Risk Consortium model, as described by Brunner *et al.* (14). A lower number/score indicates lower risk of CV disease.

^a^Age- and sex-matched untreated patients at study enrolment of NordiNet^®^ IOS or the ANSWER Program before initiating GH treatment

^b^Statistical comparison was only performed between treated and matched untreated patients at Year 2 and Year 7.

CV, cardiovascular; GH, growth hormone; IOS, International Outcome Study; *n*, number of participants.

**Figure 4:**
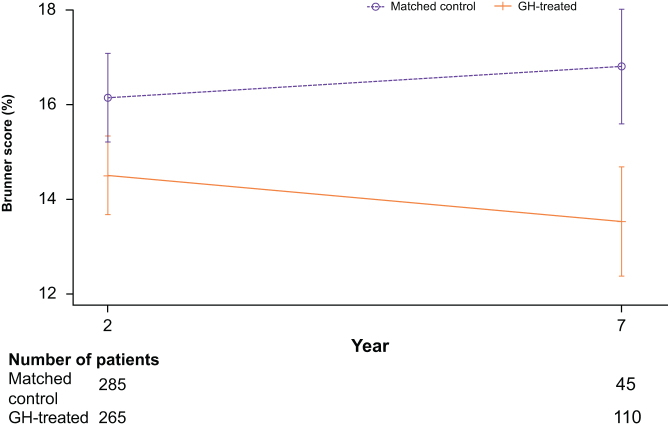
The mean (95% CI) Brunner scores of GH-treated patients and matched control groups at Year 2 and Year 7. Statistical comparisons between GH-treated and matched control patients were assessed using a Welch–Satterthwaite *t*-test due to unequal variances (Year 2: *P* = 0.0105; Year 7: *P* = 0.0001). CI, confidence interval; GH, growth hormone.

Statistical comparisons between GH-treated patients and the matched control groups confirmed statistically significant lower CV risk at Year 2 (shorter term) and Year 7 (longer term) for GH-treated patients. After 2 years of replacement therapy, Brunner score was significantly lower, with a mean score of 14.51% (95% CI: 13.67–15.34) for GH-treated patients vs 16.15% (95% CI: 15.21–17.09; *P* = 0.0105) in the matched control group (Fig. 4). After 7 years, the mean score was statistically significantly lower for GH-treated patients (13.53% (95% CI: 12.37–14.69)) than the matched control group (16.81% (95% CI: 15.60–18.02); *P* = 0.0001; Fig. 4).

The results of the sensitivity analyses showed similar trends of reduced CV risk in GH-treated patients compared with matched control groups. For patients with data available for at least three risk markers, the differences in Brunner score between GH-treated and matched controls remained statistically significant at Years 2 and 7 (data not shown). However, for patients with data available for four risk factors, while a similar trend was observed, the difference in Brunner score remained significant only for Year 7 (data not shown), which could be attributed to the low number of patients.

Regression analysis of the change from baseline in Brunner score for Years 1–10 showed a statistically significant negative trend for years of treatment (negative slope of −1.68547; *P* ≤ 0.0001), which was confirmed by a non-parametric test (Jonckheere–Terpstra; *P* ≤ 0.0001). Supplementary Fig. 3 is a paired longitudinal comparison showing GH-treated patients’ CV risk values for each follow-up year compared to their own baseline CV risk.

**Figure 5:**
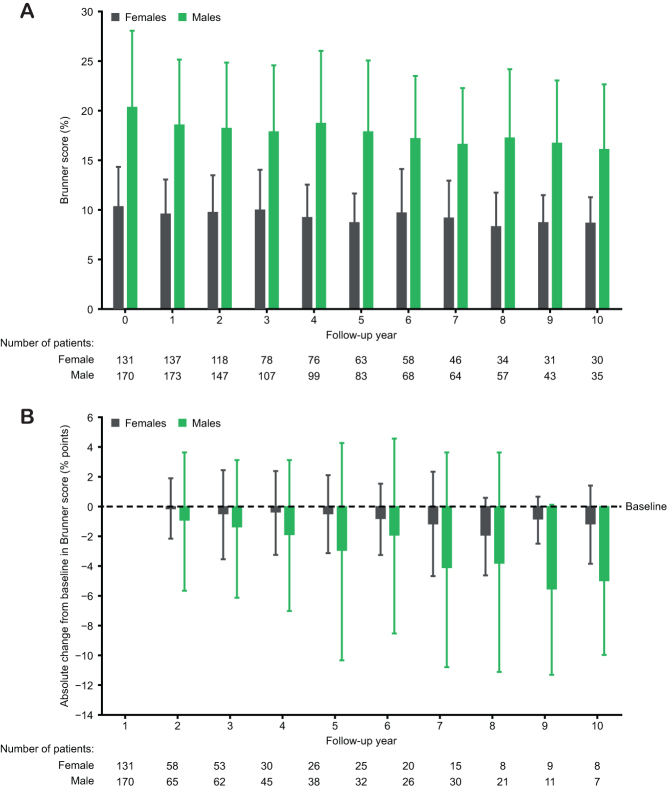
Cross-sectional and longitudinal Brunner scores of GH-treated male and female patients. (A) Mean scores and (B) absolute change from baseline. Data are mean ± s.d. GH, growth hormone.

The analysis of Brunner score by patient sex showed a greater mean (s.d.) score at baseline before GH initiation among males than females: 20.39% (7.65%) vs 10.39% (3.93%), respectively. Mean Brunner scores were reduced from baseline over 10 years in both males and females following initiation of GH replacement therapy (Fig. 5).

**Figure 6:**
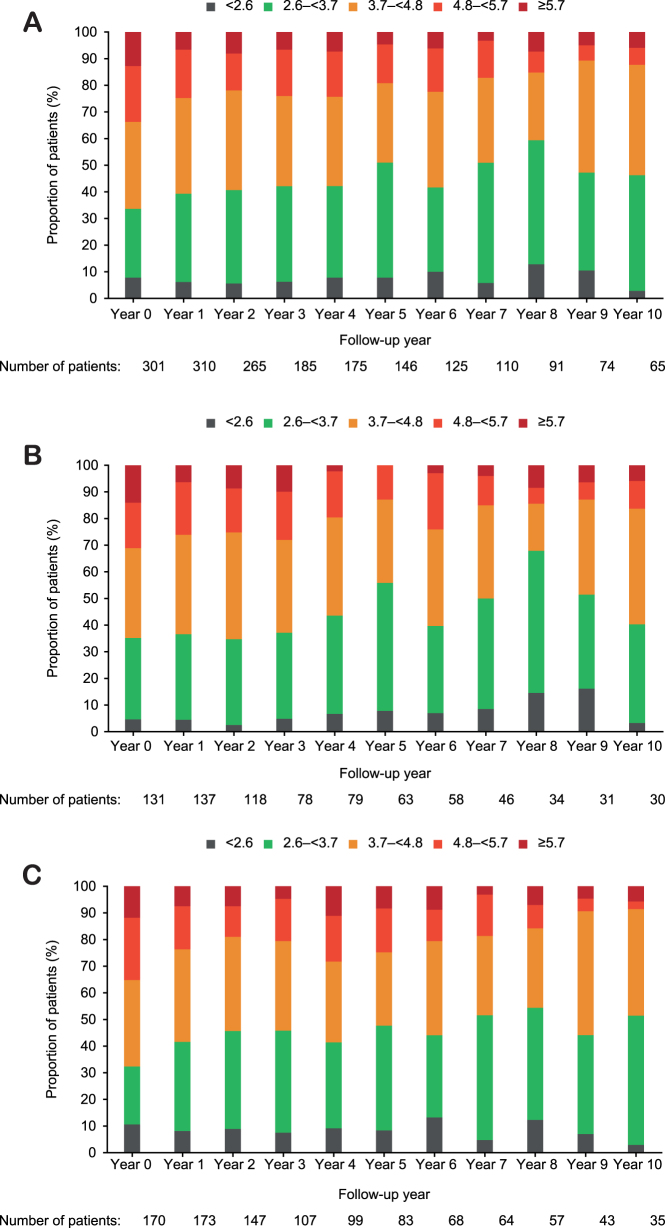
Non-HDL category (mmol/L) for GH-treated patients by follow-up year. (A) All patients, (B) females and (C) males. GH, growth hormone; HDL, high-density lipoprotein.

There were no considerable differences between GH-treated patients and the matched control groups in Year 2 or Year 7 for individual risk factors or parameters used to calculate Brunner score, except for non-HDL cholesterol, for which more GH-treated patients were within the lower categories (associated with lower CV risk) compared to matched controls. Correspondingly, more patients from the matched control groups were in the higher non-HDL categories (associated with higher CV risk) vs GH-treated patients (Table 2). The change in non-HDL cholesterol categories from baseline to Year 10 for GH-treated patients is presented in Fig. 6, showing a decreasing trend in the proportions of patients in the higher non-HDL categories (4.8 to <5.7, ≥5.7 mmol/L). A longitudinal analysis of the change from baseline in non-HDL cholesterol showed similar reductions for both the combined naïve FAS population from NordiNet^®^ IOS and the ANSWER Program and the population used in this analysis set (naïve FAS population with a Brunner score) (Supplementary Fig. 4).

Mean (s.d.) Brunner score at baseline before GH initiation was comparable among patients with presumed IGHD and MPHD: 15.69% (7.01%) vs 16.14% (8.30%). Similar reductions in Brunner score were observed in both groups over 10 years following initiation of GH replacement therapy (Supplementary Fig. 5).

In total, only 3.11% (*n*  = 22) of patients reported receiving lipid-lowering medications at baseline or during the follow-up years. Excluding these patients from the statistical comparison at Year 2 and Year 7 did not change the significance of the results. Reported use of antihypertensive and antidiabetic medications was included in the calculation for Brunner score. Sensitivity analysis assessing the effect of reported concomitant medications (thyroid hormone, corticosteroids and sex steroids) on CV risk by the age of 75 years did not change the observed decrease in Brunner score in patients treated with GH.

In total, 7 CV adverse events were reported in 6 out of the 708 patients included in this analysis. The details of these events including the patients’ Brunner score at baseline and time from start of treatment until the onset of adverse event are listed in Table 4.

**Table 4:** Reported CV events in the 708 patients included in this analysis.

Study	Patient	Sex	Age at onset	Description	SOC	PT	Serious	Causality	Onset day	Brunner score at baseline
NordiNet^®^ IOS	1	Male	71.56	Hypertension	Vascular disorders	Hypertension	No	Possible	1664	24.7
NordiNet^®^ IOS	2	Male	35.96	Heart failure	Cardiac disorders	Cardiac failure	Yes	Unlikely	1001	19.0
NordiNet^®^ IOS	3	Male	60.42	Ischemic stroke	Nervous system disorders	Ischemic stroke	Yes	Unlikely	182	31.8
NordiNet^®^ IOS	4	Male	77.21	Atrial fibrillation	Cardiac disorders	Atrial fibrillation	Yes	Unlikely	3531	12.3
NordiNet^®^ IOS	5	Female	60.12	Arrhythmia	Cardiac disorders	Arrhythmia	Yes	Unlikely	5335	8.2
NordiNet^®^ IOS	6	Male	29.58	Cardiac arrhythmia	Cardiac disorders	Arrhythmia	Yes	Unlikely	172	11.8
			29.58	Myocarditis	Cardiac disorders	Myocarditis	Yes	Unlikely	172	11.8

CV, cardiovascular; IOS, International Outcome Study; PT, preferred term; SOC, system organ class.

## Discussion

Using the Brunner score, we evaluated the risk of having CV disease by age 75 years in patients with AGHD receiving GH replacement therapy for up to 10 years and compared them with respective age- and sex-matched control groups of patients with untreated AGHD for each year. Statistical analysis for this evaluation was planned and conducted to assess the mid-term and longer-term effects of GH therapy (Years 2 and 7, respectively). The results of these cross-sectional analyses showed that GH replacement therapy is associated with a reduced risk of having CV disease by the age of 75 years in patients with AGHD who received GH replacement therapy compared with matched controls. Furthermore, comparisons between GH-treated and matched control patients at Years 2 and 7 showed a statistically significant difference, suggesting that GH replacement therapy has beneficial CV risk reduction not only in the shorter term but also in patients who continue their treatment for a longer period.

Results of the longitudinal analyses showed a gradual improvement in GH-treated patients with a decrease in Brunner score, denoting CV risk reduction, up to 10 years after the start of GH replacement therapy.

It has been reported that mortality risk is increased in untreated vs treated patients with GHD (18) and that patients with MPHD have an increased CV mortality and morbidity risk, owing to unreplaced GH, the aetiology of hypopituitarism and non-physiological replacement therapy of other hormone deficiencies (19, 20, 21). Long-term GH replacement in our study was associated with lower CV risk in both MPHD and presumed IGHD patients, which further underlines the need for GH replacement therapy in patients with MPHD and adds to available evidence suggesting the importance of GH replacement in these patients (22, 23, 24).

The effect of GH replacement therapy on several CV risk markers, including blood glucose homeostasis, BMI, waist circumference and bioimpedance in patients with AGHD from NordiNet^®^ IOS, has been previously described (10, 17). The effect on glucose homeostasis was reported in 245 nondiabetic patients with adult-onset GHD who received Norditropin^®^ (Novo Nordisk A/S, Denmark) for at least 4 years (10). The mean baseline glycated haemoglobin (HbA1c) was 32.6 mmol/mol (5.13%) in that study and remained the same after 4 years. Additionally, most patients (85%) who were within the normal range at baseline were still within the normal range after 4 years, while 55% of patients with impaired glucose tolerance at baseline remained in this category after 4 years. Furthermore, a similar effect of GH replacement on glucose homeostasis and the potential beneficial effect on lipid profile after GH replacement therapy, with a reduction in total and LDL cholesterol and an increase in HDL cholesterol, has been observed in other long-term studies (12, 25, 26, 27, 28). Thus, long-term GH replacement therapy is associated with an improvement in body composition and metabolic parameters that may reduce CV risk (17, 29, 30).

In our study, additional analysis suggested that changes in non-HDL cholesterol may be a major contributor to the observed decrease in Brunner score in GH-treated patients. No considerable differences were observed between GH-treated patients and the matched control groups in Years 2 and 7 for the risk factors used to calculate Brunner score except for non-HDL cholesterol. More GH-treated patients were within the lower non-HDL cholesterol categories vs matched controls. These results are in line with the observed statistically significant lower Brunner scores in GH-treated patients.

The results from our study corroborate those of a prospective study of the German cohort of the Pfizer International Metabolic Database (KIMS) that estimated CV risk in patients with AGHD compared with healthy controls using the Framingham index, Prospective Cardiovascular Münster Heart Study (PROCAM) and European Society of Cardiology score algorithms (8). After 2 years of GH replacement therapy, the risk scores for all three algorithms were approximately 50% lower compared with baseline and returned to baseline levels after 4 years. The risk after 4 years, however, was significantly higher in healthy controls (8). The Framingham index was also used to assess CV risk in patients with AGHD from the Italian cohort of the observational Hypopituitary Control and Complication Study (HypoCSS). Patients were grouped by duration of treatment: ≤2 years (*n*  = 451), >2 to ≤6 years (*n*  = 387) and >6 years (*n*  = 395) (31). The Framingham index decreased for patients with the shortest duration of GH treatment and increased for the other two groups; the authors hypothesised that the increase in risk may be due to the increased age of patients in the longer duration groups (31).

A strength of this study is the large patient population. We compared the estimated risk of CV disease in GH-treated patients with age-and sex-matched controls (patients with untreated AGHD), adding to the observations of KIMS and HypoCSS. The long-term follow-up (10 years) of patients, as well as the longitudinal comparison showing a clear trend over time, can be considered as other strengths of our study. The observational setting was advantageous since it allowed both the inclusion of a paired control group and assessment of the beneficial effect of longitudinal GH replacement on CV risk via long-term observation. Finally, our study shows an association and does not prove causality but, due to the congruent data, it is strongly suggestive of a beneficial effect of GH replacement therapy on CV risk in patients with AGHD.

There are, however, several limitations to this study. NordiNet^®^ IOS and the ANSWER Program were subject to the general limitations of large multicentre observational studies, for which there may have been underreporting of comorbidities, concomitant medications and adverse events. Potential underreporting of concomitant medications may have masked their true impact on the risk of CV disease by the age of 75 years and underreporting of comorbidities could have contributed to the missing risk factor data required for calculating the Brunner score. A combined approach to categorise the data required for the classification of risk factors was implemented to mitigate this limitation, as explained in the ‘Variables and statistical analysis’ section. The validity of this approach was confirmed by the results of the two sensitivity analyses conducted. Similarly, the potential underreporting of comorbidities could have affected the number of reported pituitary deficiencies at baseline. This could explain the higher percentage of patients with presumed IGHD in our study population compared with other studies (8). A decreasing number of patients were captured in each subsequent year of follow-up, which is typical of longitudinal observational studies. Both NordiNet^®^ IOS and the ANSWER Program continued to enrol patients throughout their duration, meaning that some patients were enrolled toward the end of the study and had limited follow-up data. Furthermore, the data regarding the time from diagnosis of AGHD to starting GH replacement therapy are not captured for all patients within NordiNet^®^ IOS and the ANSWER Program and, accordingly, accurate duration of the disease could not be matched between the GH-treated and control groups. Each population with a specific number of years of follow-up may be considered as not fully representative of the general AGHD population. We have tried to limit the potential impact of this by comparing treated patients with age- and sex-matched controls both at shorter- and longer-term follow-up. We planned statistical comparisons at Year 2 and Year 7, which were before and after the mean follow-up year of the original studies (17), to address the concern of a possible selection bias for the patients with a follow-up duration longer than that of the mean.

Furthermore, comparable non-HDL cholesterol levels in the total naïve FAS population from the combined NordiNet^®^ IOS and ANSWER Program studies and our study subjects (naïve FAS population with sufficient data available to calculate Brunner score) suggest that our approach of accounting for missing risk factor data had minimal to no impact on the results. Another limitation may have been the fact that parameters were measured by local laboratories rather than in a central facility. As the Multinational CV Risk Consortium model was developed using data from individuals without prevalent CV disease, and the model is applied in the current study to a population of patients with an inherently higher CV risk, the results should be interpreted with caution; GHD is associated with increased fat mass and an abnormal lipid profile, which may contribute to the excess CV mortality observed in patients with AGHD (9). Finally, although our long-term follow-up data suggest improved non-HDL cholesterol levels and reduced CV risk over 10 years of follow-up, the observational nature of the study and relatively low number of patients in the later follow-up years should be considered in interpretation of the results. A prospective study measuring traditional CV endpoints is needed to further elucidate the impact of GH treatment on CV risk.

In conclusion, results from our analyses suggest that GH replacement therapy in patients with AGHD may reduce CV disease risk by the age of 75 years, as assessed by calculated Brunner score, compared with age- and sex-matched controls. The risk reduction appears to be sustained over time, further supporting the continued benefits of long-term use of GH replacement therapy in these patients.

## Supplementary Material

Supplementary Material

## Declaration of interest

CH has received honoraria for lectures and consultation from Novo Nordisk, Pfizer, Ascendis and Sandoz and is a member of the Global Steering Committee for the PATRO Adults study. BMKB has been the principal investigator of a research grant from Ascendis to Massachusetts General Hospital and has received occasional consulting honoraria from Ascendis, Merck-Serono and Novo Nordisk. JMF is a consultant for Novo Nordisk. MBG has received research support from Chiasma, Corcept, Crinetics, Ipsen, Lilly, Novartis, Pfizer, Recordati and Strongbridge and has received honoraria for consultation from HRA Pharma, Ipsen, Novo Nordisk and Recordati. AHO is an employee of and stockholder in Novo Nordisk. NK is an employee of Novo Nordisk and a stockholder in Novo Nordisk and Pfizer. NN is an employee of Novo Nordisk. MW has received consulting and speaker honoraria from Ipsen, Lilly, Recordati and Novo Nordisk.

## Funding

This work was supported by Novo Nordisk http://dx.doi.org/10.13039/501100004191 Health Care AG (for statistical analysis and editorial support).
