# Supplementary material for: Reduced CV risk with long-term GH replacement in AGHD: data from two large observational studies
Source: Endocr Connect. 2022 Dec 14;12(1):e220267. doi: 10.1530/EC-22-0267 (PMC9782424; doi:10.1530/EC-22-0267)
Supplement: Supplementary Material [file supplementary_material.pdf]

Reduced CV risk with long-term GH replacement in AGHD: data from two large observational studies

Charlotte Höybye, Beverly M K Biller, Jean-Marc Ferran, Murray B Gordon, Nicky Kelepouris, Navid Nedjatian, Anne H Olsen and Matthias M Weber

Supplementary Appendix

Materials and methods

Patient population

The **full analysis set** (FAS) included all growth hormone (GH)-naïve and non-naïve patients with a diagnosis of growth hormone deficiency (GHD) who started GH replacement therapy after the age of 18 years and who were treated with GH after the age of 20 years. As per protocol of the two studies, paediatric patients could be treated to final height after the age of 18 years. Patients with Norditropin® (somatropin; Novo Nordisk A/S, Denmark) treatment initiation after the age of 18 years and no Norditropin® treatment after the age of 20 years were instead included in the paediatric FAS and are not included in this analysis.

Variables and statistical analysis

For GH-treated patients, age at a given year was defined as age at start of treatment plus the number of years of treatment. Non-high-density lipoprotein (HDL) cholesterol was defined as total cholesterol minus HDL cholesterol. These laboratory parameters in both NordiNet® IOS and the ANSWER Program were measured according to routine clinical practice and no central laboratory was used. Patients were identified as hypertensive when their systolic blood pressure (SBP) was ≥140 mmHg or when their SBP was <140 mmHg with reported concomitant use of an antihypertensive medication. Patients were considered positive for daily smoking if they were identified as a regular smoker in the lifestyle section of the study case reports. Patients were identified as having diabetes when their fasting plasma glucose (FPG) was ≥126 mg/dL, or their HbA1c was ≥47.5 mmol/mol (6.5%), or when either FPG or HbA1c were below the defined threshold with reported concomitant use of antidiabetic medications. Patients were identified as obese if their body mass index (BMI) was ≥30 kg/m^2. Patients were classified as having 0–1 cardiovascular risk factor or ≥2 risk factors.

Years 2 and 7 were selected to address the potential difference between patients with shorter-term follow-up and those with longer-term follow-up. Year 7 was also the last year in which at least 100 treated patients were available for analysis, as the number of patients fell further in the subsequent years.

Change in Brunner score over 10 years following GH replacement initiation was also analysed by patient sex (male or female), and the number of reported pituitary deficiencies (presumed isolated growth hormone deficiency [presumed IGHD] or multiple pituitary hormone deficiency [MPHD]). The results are presented descriptively. Sensitivity analysis was performed to assess the effect of reported concomitant medications (thyroid hormone, corticosteroids, sex steroids) on the observed changes in cardiovascular risk.

Supplementary Figure 1. The Multinational Cardiovascular Risk Consortium model (Brunner score) for prediction of risk of CV disease by 75 years of age, using non-HDL cholesterol categories adjusted for age, sex, cohort, and modifiable CV risk factors, adapted from Brunner *et al*. 2019 (1).

Age, years	Number of risk factors*	Non-HDL cholesterol, mmol/L <2.6	2.6–<3.7	3.7–<4.8	4.8–<5.7	≥5.7
**Female**						
		Risk score	Risk score	Risk score	Risk score	Risk score
<45	0–1	5.7	6.9	8.8	10.9	14.0
	≥2	12.3	13.4	15.6	18.1	24.1
45–59	0–1	5.6	6.7	8.2	10.0	12.9
	≥2	11.3	12.8	14.6	16.9	22.1
≥60	0–1	5.0	5.7	6.7	8.2	10.7
	≥2	9.1	9.8	11.9	13.8	17.7
**Male**						
Age, years	Number of risk factors*	Non-HDL cholesterol, mmol/L <2.6	2.6–<3.7	3.7–<4.8	4.8–<5.7	≥5.7
		Risk score	Risk score	Risk score	Risk score	Risk score
<45	0–1	11.8	15.0	19.0	23.4	29.8
	≥2	18.9	24.2	28.8	33.4	43.0
45–59	0–1	10.6	13.2	16.4	20.3	27.0
	≥2	19.3	23.0	27.0	31.8	40.9
≥60	0–1	7.8	9.9	12.3	14.8	19.6
	≥2	15.5	17.2	21.0	24.7	31.6

*Hypertension, daily smoking, diabetes, and obesity.

CV, cardiovascular; HDL, high-density lipoprotein.

Supplementary Figure 2. Cross-sectional comparison of IGF-I SDS between GH-treated male and female patients at a follow-up year and age- and sex-matched controls.

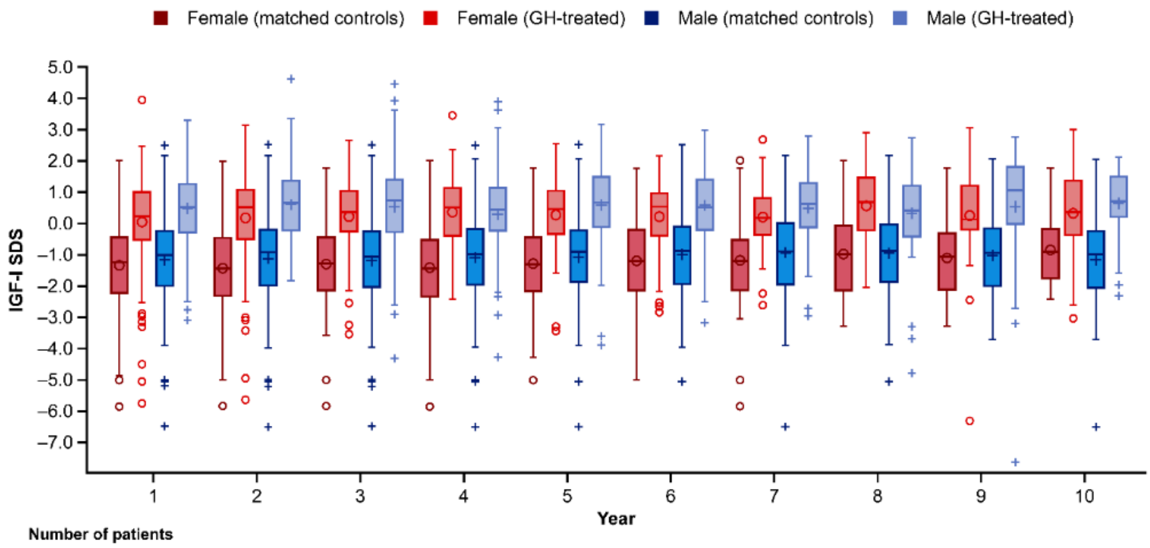

Number of patients

	1	2	3	4	5	6	7	8	9	10
**Matched controls**										
Female	107	123	103	105	95	79	71	55	56	49
Male	145	151	144	136	119	106	101	92	70	57
**GH-treated**										
Female	135	115	78	75	62	58	45	34	31	28
Male	167	145	105	98	81	67	64	57	43	34

Shown are the mean (*noughts/crosses within the box*), median (*dash within the box*), 25th/75th percentiles (*box*) and maximum overserved values (*noughts and crosses above the box*).

GH, growth hormone; IGF-I, insulin-like growth factor-I; SDS, standard deviation score.

Supplementary Figure 3. Longitudinal comparison of Brunner score at baseline and follow-up year by year for GH-treated patients.

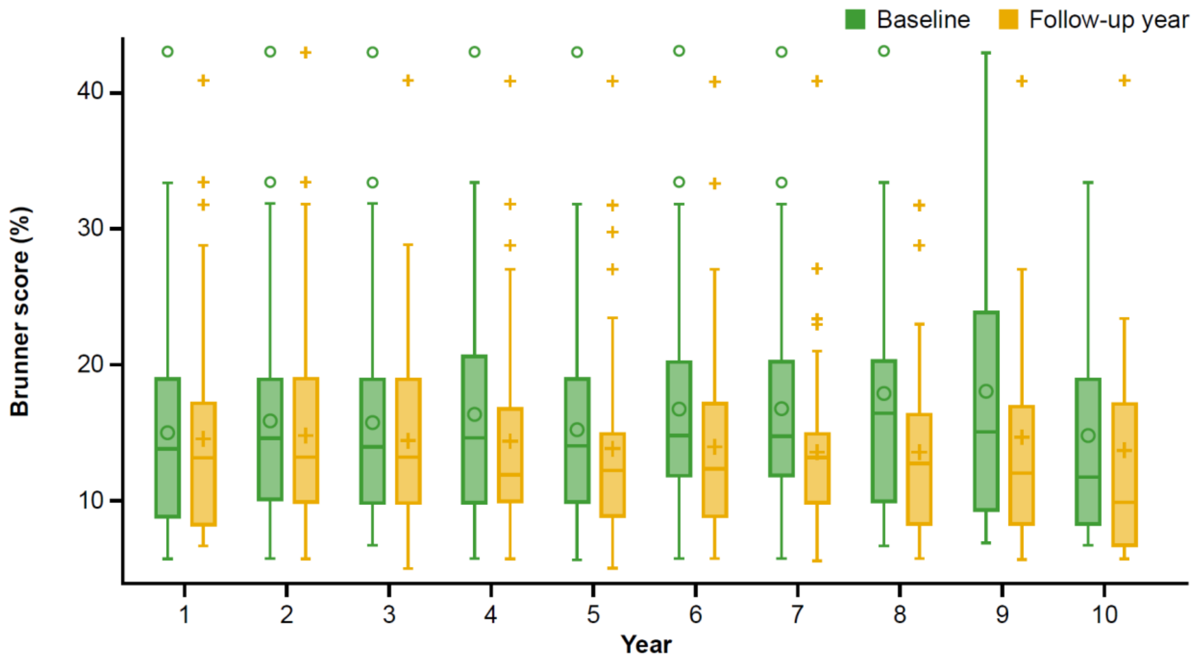

Number of patients

Baseline	123	115	75	64	57	46	45	29	20	15
Follow-up year	123	115	75	64	57	46	45	29	20	15

Shown are the mean (*noughts/crosses within the box*), median (*dash within the box*), 25th/75th percentiles (*box*) and maximum overserved values (*noughts and crosses above the box*).

Regression analysis of the change from baseline in Brunner score for years 1–10 was confirmed by a non-parametric test (Jonckheere-Terpstra; $P \leq 0.0001$).

GH, growth hormone.

Supplementary Figure 4. Change from baseline in non-HDL cholesterol for ‘naïve patients (FAS)’ and ‘naïve patients with a Brunner score (FAS)’ by follow-up year. **A)** females, **B)** males.

A

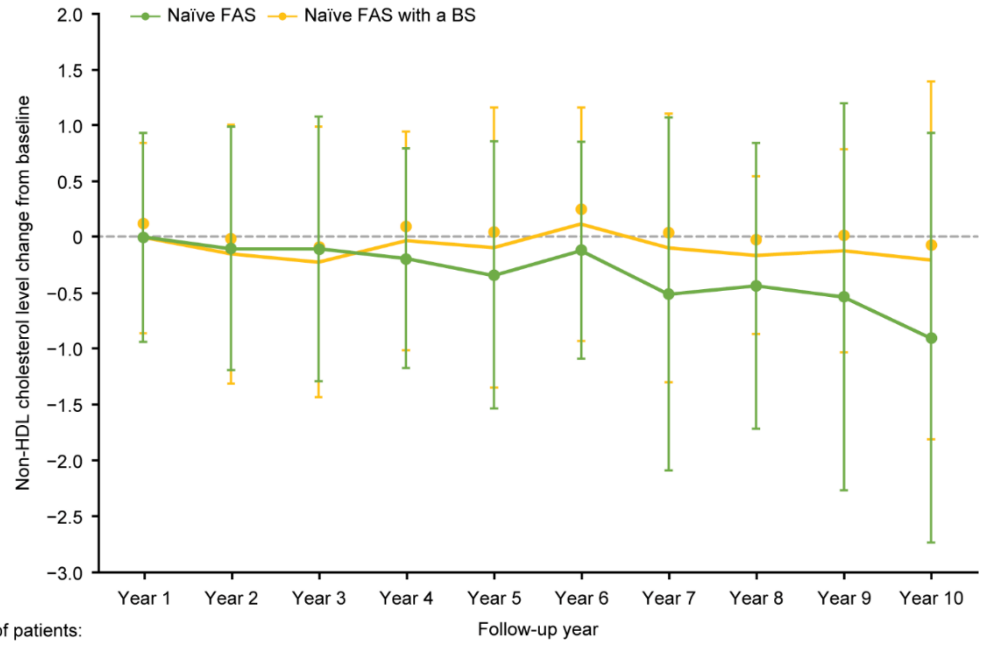

Number of patients:										
Naïve FAS	141	105	85	73	66	50	36	26	28	18
Naïve FAS with a BS	58	53	30	26	25	20	15	8	9	8

B

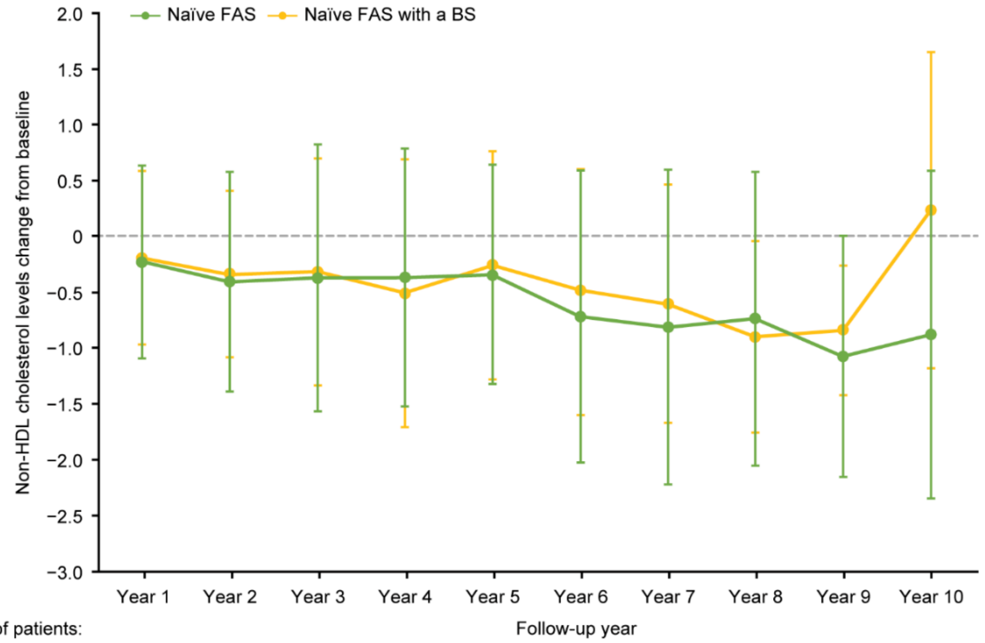

Number of patients:										
Naïve FAS	172	154	112	88	72	61	56	40	34	24
Naïve FAS with a BS	65	62	45	38	32	26	30	21	11	7

Data are mean (SD).

FAS, full analysis set; GH, growth hormone; HDL, high-density lipoprotein.

Supplementary Figure 5. Cross-sectional and longitudinal Brunner scores of GH-treated patients with presumed IGHD and MPHD. **A)** Mean scores, **B)** absolute change from baseline.

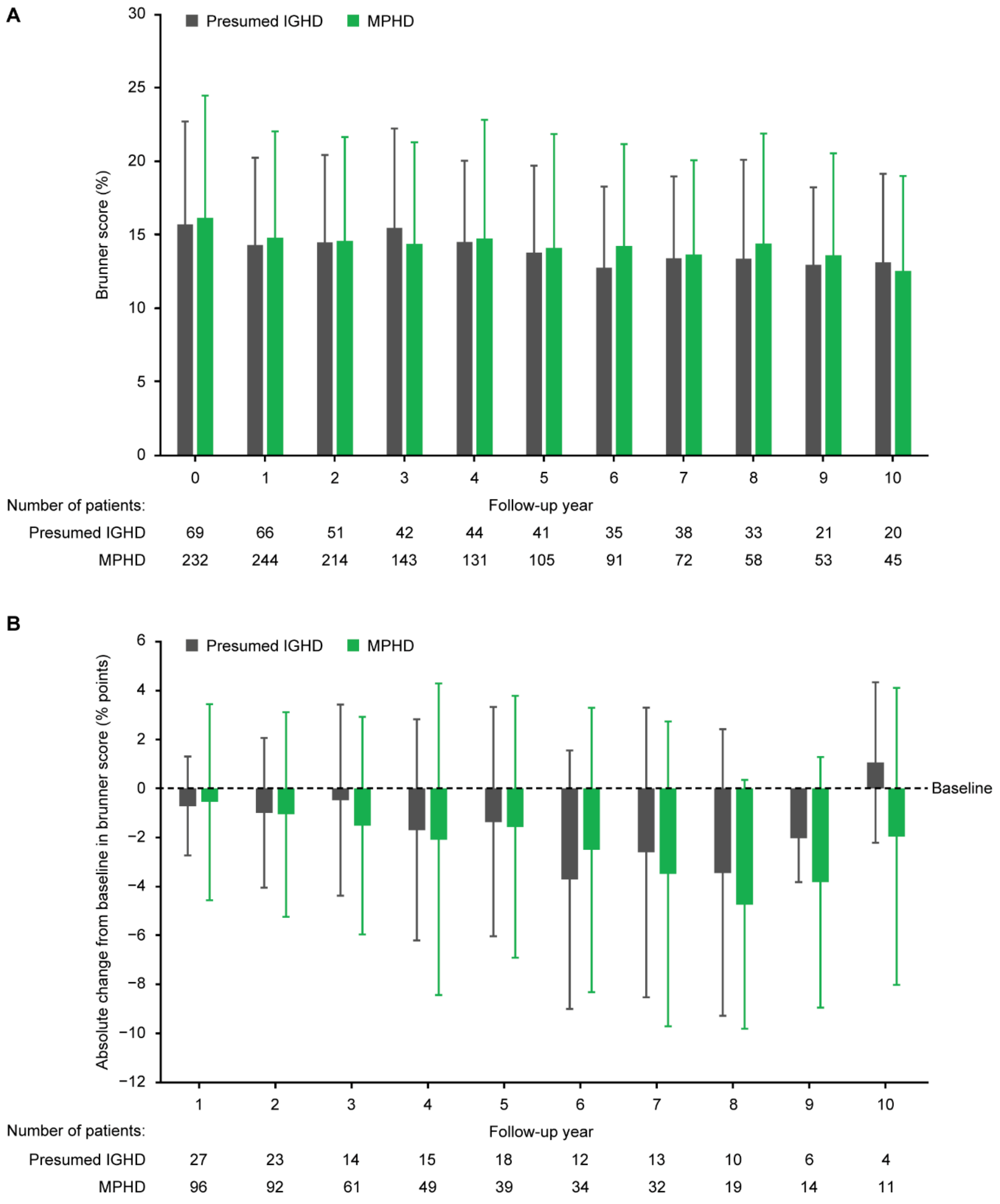

Data are mean ± SD.

GH, growth hormone; presumed IGHD, presumed isolated growth hormone deficiency; MHPD, multiple pituitary hormone deficiency; SE, standard deviation.

Reference

1. Brunner FJ, Waldeyer C, Ojeda F, Salomaa V, Kee F, Sans S, Thorand B, Giampaoli S, Brambilla P, Tunstall-Pedoe H, Moitry M, Iacoviello L, Veronesi G, Grassi G, Mathiesen EB, Soderberg S, Linneberg A, Brenner H, Amouyel P, Ferrieres J, Tamosiunas A, Nikitin YP, Drygas W, Melander O, Jockel KH, Leistner DM, Shaw JE, Panagiotakos DB, Simons LA, Kavousi M, Vasan RS, Dullaart RPF, Wannamethee SG, Riserus U, Shea S, de Lemos JA, Omland T, Kuulasmaa K, Landmesser U, Blankenberg S & Multinational Cardiovascular Risk Consortium. Application of non-HDL cholesterol for population-based cardiovascular risk stratification: results from the Multinational Cardiovascular Risk Consortium. *Lancet* 2019 **394** 2173–2183.
